# Adjuvant targeted therapy combined with surgery for advanced and metastatic renal cell carcinoma: A protocol for systematic review and meta analysis

**DOI:** 10.1097/MD.0000000000023956

**Published:** 2021-01-22

**Authors:** Hongyu Jin, Man Zhang, Kun Jin, Chenggong Hu

**Affiliations:** a Department of Liver Surgery, Liver Transplantation Center, West China Hospital, Sichuan University, Chengdu; b Department of Gynecology and Obstetrics, West China Second University Hospital, Sichuan University; Key Laboratory of Obstetric & Gynecologic and Pediatric Disease and Birth Defects of Ministry of Education; c Department of Urology, Institute of Urology; d Department of Critical Care Unit, West China Hospital, Sichuan University, Chengdu, China.

**Keywords:** adverse effects, efficacy, perioperative use of sunitinib, safety

## Abstract

**Background:**

The aim of this systematic review and meta-analysis is to evaluate the efficacy and safety of adjuvant targeted therapy by sunitinib combined with surgery in the treatment of advanced or metastatic renal cell carcinoma.

**Methods:**

PubMed/Medline, Web of Science, Cochrane Library, ClinicalTrials.gov (http://www.ClinicalTrials.gov), China National Knowledge Infrastructure (CNKI) will be searched for clinical research articles related to the efficacy and safety of adjuvant therapy combined with surgery in the treatment of advanced and metastatic RCC. The identification, inclusion and exclusion flow charts will be conducted according to the PRISMA guidelines. The quality assessment will be done by Quadas-2 evaluation tool. Key parameters including OS in 10, 20, 30, and 40 months, PFS in 10, 20, and 30 months, objective response rate (ORR), stable disease (SD) rate, progressive disease (PD) rate, median OS and PFS, types of AEs and their occurrence rates, etc will be extracted. The evaluation of the efficacy and safety will be pooled by CMA.

**Results:**

This systematic review will provide evidence on the efficacy and safety of adjuvant therapy by sunitinib combined with surgery in treating advanced and metastatic RCC.

**Conclusion:**

The study aims to generalize data concerning the response rate, OS, PFS and rates of adverse effects of the perioperative use of sunitinib in advanced and metastatic RCC patients. The evidence provided by this systematic review and meta-analysis will help guide the clinical decision making and enlighten the future management of advanced or metastatic RCC.

**Registration:**

This protocol has been registered on the International Platform of Registered Systematic Review and Meta-analysis Protocols (INPLASY registration number: INPLASY2020110093; INPLASY DOI number: 10.37766/inplasy2020.11.0093 Available at: https://inplasy.com).

## 1 Introduction

Renal cell carcinoma (RCC) is one of the most common malignancies of the genitourinary tract originating from the cells in proximal convoluted tubule.^[1,2]^ Accordingly, the global mortality nearly doubled in 15 years from 1985 to 2000.^[3,4]^ Clinically, the majority of patients are asymptomatic during the early stages of RCC, thus only a fraction of patients is efficiently diagnosed and subsequently managed.^[5]^ Due to the rapid progress and high invasiveness of RCC, patients who are only correctly managed during late stage have a comparatively low possibility of complete recovery.^[6,7]^

One of the most important factors contributing to the rapid development and invasiveness is its tendency of metastasis.^[8]^ Specifically, direct metastasis into the peritoneal cavity, migration into the blood vessels and even formation of large-sized venous thrombus into the right atrial system are common pathways for disease progress.^[9,10]^ Usually, the appearance of venous thrombus in the right atrial system marks the turning point for the escalation of disease severity.^[11]^ Despite knowing the rigorous fact, solutions to effectively remove the lesions are still limited. Currently, surgery is the first choice for patients diagnosed with advanced conditions like this for it directly removes both the original tumor and the metastatic thrombus.^[12]^ Nevertheless, radical surgeries often involve sternotomy and optional cardiac arrest, which are challenging. As a result, adjuvant drug therapy has increasingly been adopted to aid surgical process or to directly treat patients.^[13,14]^

Sunitinib is a tyrosine kinase inhibitor (TKI) which is usually used in adjuvant targeted therapy.^[15]^ It can inhibit several receptors which have an effect in tumorigenesis and tumor progress of RCC, including vascular endothelial growth factor receptors (VEGFRs, like VEGFR-1, VEGFR-2, VEGFR-3) and c-Kit.^[16,17]^ Angiogenesis is mandatory in the progression of RCC since it provides tumor tissues with adequate oxygen and nutrients. Thus, combined use of TKIs which helps to restrict the emerging of blood vessels is believed to play a role in managing RCC.

Up to now, several important clinical trials have been carried out worldwide to evaluate the efficacy and safety of the aforementioned combined adjuvant targeted therapy and surgery. Some studies including 2 famous landmark trials have pointed out that this combined therapy could degrade tumor stage and halt tumor development and reduce the size of both the original tumor and metastatic neoplasms.^[18,19]^ However, there exist other reports claiming that such combination had no direct impact on tumor growth and on the contrary would bring other drug safety concerns such as provoking several adverse effects. These mainly included hand and foot syndrome, malaise, digestive problems, etc. Besides, many of these studies are single-arm trials or fail to recruit sufficient number of patients. Therefore, the present study will synthesize the results of current available evidences to assess the efficacy and safety of the combined therapy of surgery and adjuvant targeted therapy in patients with advanced or metastatic RCC.

## 2 Material and methods

This protocol has been registered on the International Platform of Registered Systematic Review and Meta-analysis Protocols (INPLASY registration number: INPLASY2020110093; INPLASY DOI number: 10.37766/inplasy2020.11.0093 Available at: https://inplasy.com). This protocol will be conducted according to the guidelines of Preferred Reporting Items for Systematic Reviews and Meta-Analysis Protocols (PRISMA-P). This study has been approved by the Ethics Committee of West China Hospital, Sichuan University (Chengdu China).

### 2.1 Search strategy

Following PRISMA-P, we will carefully search authenticated databases including PubMed/Medline, Web of Science, Cochrane Library, ClinicalTrials.gov (http://www.ClinicalTrials.gov), China National Knowledge Infrastructure (CNKI) for clinical research articles related to the efficacy and safety of adjuvant therapy combined with surgery in the treatment of advanced and metastatic RCC. In order to study the latest articles, we will only include articles published between January 2008 and May 2020.

### 2.2 Article selection

We will assign 3 independent reviewers to judge duplicates and relevancy, in which obvious duplicates and irrelevant articles will be excluded from further analysis. Remaining articles will be interrogated to extract full texts and raw data. In this process, case reports, reviews, letters and meeting proceedings will be excluded.

The inclusion criteria will include: first, reported at least either indicators for survival analysis or data concerning the AEs; and second, randomized controlled trials and any observational design, including cross-sectional, case-control, and cohort designs.

The consensus on the article selection process will be reported and more independent reviewers will be consulted if discrepancy should occur.

### 2.3 Data extraction

After selecting qualified articles, we will extract important information from these articles. The information will mainly include the basic details of the articles, patients demographic characteristics, data concerning the efficacy and safety. More specifically, key parameters will include OS in 10, 20, 30, and 40 months, PFS in 10, 20, and 30months, objective response rate (ORR), stable disease (SD) rate, progressive disease (PD) rate, median OS and PFS, types of AEs and their occurrence rates, etc. The baseline characteristics of the articles will include title, first author, nationality, department, study design and enrollment year. Finally, sex and median age, ethnicity of the patients will also be carefully extracted as the demographic features.

### 2.4 Quality assessment

We will perform standard quality assessment of the included studies based on Quadas-2 tool. By Quadas-2, the articles will be evaluated in the following processes: sequence generation (selection bias), allocation concealment (selection bias), blinding of participants and personnel (performance bias), blinding of outcome assessment (detection bias), incomplete outcome data (attrition bias), selective reporting (reporting bias), and others.

### 2.5 Publication bias

To avoid publication bias, if over 10 articles will be included to extract qualified data for further analysis, we will apply the “funnel plot” to detect the potential risk of publication bias. If not, we will implement Begg test and Egger test. All the aforementioned tests will be performed through Stata 14.2 (Stata Corp).

### 2.6 Heterogeneity assessment

We will apply *I*^2^ statistics and Galbraith plot method to evaluate the heterogeneity. If *I*^2^ < 50%, we will use a fixed-effects model. If we come across a high heterogeneity, we will use the Galbraith plot to identify the outliers and thus perform a sensitivity analysis.

### 2.7 Statistical analysis

The occurrence rate of AEs, including AEs of all grades and of grade ≥3 AEs as well as their 95% confidential interval (CIs) will be calculated based on data collected from these single-arm trials. All the analyses and calculations mentioned above will be conducted using comprehensive meta-analysis (CMA) (Biostat, Englewood, NJ).

## 3 Discussion

Advanced and metastatic RCC is a common type of malignancy of the kidney which easily leads to life-threatening events. The first-line treatment of advanced and metastatic RCC is radical surgery, despite that the surgery itself has quite a high degree of risk.^[20]^ To provide better prognostic opportunity, physicians have brought up the concept of combined adjuvant therapy by sunitinib. Although a series of studies proved that adjuvant therapy by sunitinib was beneficial in that it was able to decrease tumor size and prevent continuous production of tumor thrombus.^[21]^ However, there were indeed some other studies providing the opposite conclusions. This systematic review and meta-analysis will objectively report the efficacy of sunitinib based on large data analysis.^[22]^

Besides the efficacy, the safety concerns of sunitinib were also heatedly discussed. So far, previous articles have claimed that proteinuria, anemia, asthenia, pause syndromes, etc were the most common AEs. Through systematic review and meta-analysis, we will provide the general pooled occurrence rate of all-grade AEs and grade≥3 AEs in order to enlighten the safety concerns of sunitinib.

## Author contributions

**Conceptualization:** Hongyu Jin, Chenggong Hu.

**Data curation:** Hongyu Jin, Man Zhang.

**Formal analysis:** Hongyu Jin, Kun Jin.

**Funding acquisition:** Chenggong Hu.

**Investigation:** Hongyu Jin, Man Zhang.

**Methodology:** Man Zhang.

**Project administration:** Hongyu Jin.

**Resources:** Hongyu Jin, Chenggong Hu.

**Software:** Hongyu Jin, Man Zhang, Kun Jin.

**Validation:** Hongyu Jin, Man Zhang.

**Visualization:** Hongyu Jin, Kun Jin.

**Writing – original draft:** Hongyu Jin, Man Zhang.

**Writing – review & editing:** Hongyu Jin, Chenggong Hu.

## References

[1] Motzer RJ Rini BI McDermott DF Nivolumab plus ipilimumab versus sunitinib in first-line treatment for advanced renal cell carcinoma: extended follow-up of efficacy and safety results from a randomised, controlled, phase 3 trial. Lancet Oncol 2019;20:1370–85. 31427204 10.1016/S1470-2045(19)30413-9 PMC7497870

[2] Czarnecka AM Sobczuk P Korniluk J Long-term response to sunitinib: everolimus treatment in metastatic clear cell renal cell carcinoma. Future Oncol (London, England) 2017;13:31–49. 10.2217/fon-2016-0355 27599260

[3] Sun M Marconi L Eisen T Adjuvant vascular endothelial growth factor-targeted therapy in renal cell carcinoma: a systematic review and pooled analysis. Eur Urol 2018;74:611–20. 29784193 10.1016/j.eururo.2018.05.002 PMC7515772

[4] Tornberg SV Visapaa H Kilpelainen TP Surgery for metastases of renal cell carcinoma: outcome of treatments and preliminary assessment of Leuven-Udine prognostic groups in the targeted therapy era. Scandinavian J Urol 2018;52:419–26. 10.1080/21681805.2018.1553893 30663485

[5] Zhang G Wu Y Zhang J Nomograms for predicting long-term overall survival and disease-specific survival of patients with clear cell renal cell carcinoma. Onco Targets Ther 2018;11:5535–44. Wood SY, Ryan JC, Clair AG, George DJ: Understanding the adverse event experience in the S-TRAC adjuvant trial of sunitinib for high-risk renal cell carcinoma. *Future oncology (London, England)* 2020. 30233214 10.2147/OTT.S171881 PMC6134959

[6] Guida FM Santoni M Conti A Alternative dosing schedules for sunitinib as a treatment of patients with metastatic renal cell carcinoma. Critical Rev Oncol/Hematol 2014;92:208–17. 10.1016/j.critrevonc.2014.07.006 25151214

[7] Escudier B Choueiri TK Oudard S Prognostic factors of metastatic renal cell carcinoma after failure of immunotherapy: new paradigm from a large phase III trial with shark cartilage extract AE 941. J Urol 2007;178:1901–5. 17868728 10.1016/j.juro.2007.07.035

[8] Xu W Puligandla M Manola J Angiogenic factor and cytokine analysis among patients treated with adjuvant VEGFR TKIs in resected renal cell carcinoma. Clin Cancer Res 2019;25:6098–106. 31471309 10.1158/1078-0432.CCR-19-0818 PMC7057142

[9] Haferkamp A Bastian PJ Jakobi H Renal cell carcinoma with tumor thrombus extension into the vena cava: prospective long-term followup. J Urol 2007;177:1703–8. 17437789 10.1016/j.juro.2007.01.039

[10] Schueler S Schuetz GM Dewey M The revised QUADAS-2 tool. Ann Intern Med 2012;156:323 author reply 323–324. 22351721 10.7326/0003-4819-156-4-201202210-00018

[11] Amin C Wallen E Pruthi RS Preoperative tyrosine kinase inhibition as an adjunct to debulking nephrectomy. Urology 2008;72:864–8. 18684493 10.1016/j.urology.2008.01.088

[12] Fujita T Hirayama T Ishii D Efficacy and safety of sunitinib in elderly patients with advanced renal cell carcinoma. Mol Clin Oncol 2018;9:394–8. 30214728 10.3892/mco.2018.1684 PMC6125697

[13] Amin A Plimack ER Ernstoff MS Safety and efficacy of nivolumab in combination with sunitinib or pazopanib in advanced or metastatic renal cell carcinoma: the Check Mate 016 study. J Immunotherapy Cancer 2018;6:109. 10.1186/s40425-018-0420-0 PMC6196426 30348216

[14] Escudier B Roigas J Gillessen S Phase II study of sunitinib administered in a continuous once-daily dosing regimen in patients with cytokine-refractory metastatic renal cell carcinoma. J Clin Oncol 2009;27:4068–75. 19652072 10.1200/JCO.2008.20.5476

[15] Bex A Powles T Karam JA Role of targeted therapy in combination with surgery in renal cell carcinoma. Int J Urol 2016;23:5–12. 26238981 10.1111/iju.12891

[16] Margulis V Matin SF Tannir N Surgical morbidity associated with administration of targeted molecular therapies before cytoreductive nephrectomy or resection of locally recurrent renal cell carcinoma. J Urol 2008;180:94–8. 18485389 10.1016/j.juro.2008.03.047

[17] Brehmer B Kauffmann C Blank C Resection of metastasis and local recurrences of renal cell carcinoma after presurgical targeted therapy: probability of complete local control and outcome. World J Urol 2016;34:1061–6. 27287888 10.1007/s00345-016-1865-8

[18] Heng DY Xie W Regan MM External validation and comparison with other models of the International Metastatic Renal-Cell Carcinoma Database Consortium prognostic model: a population-based study. Lancet Oncol 2013;14:141–8. 23312463 10.1016/S1470-2045(12)70559-4 PMC4144042

[19] Gleeson JP Motzer RJ Lee CH The current role for adjuvant and neoadjuvant therapy in renal cell cancer. Curr Opinion Urol 2019;29:636–42. Escudier B, Szczylik C, Porta C, Gore M: Treatment selection in metastatic renal cell carcinoma: expert consensus. *Nature reviews Clinical oncology* 2012, 9(6):327-337. 10.1097/MOU.0000000000000666 31348025

[20] Hekman MC, Rijpkema M, Muselaers CH, Oosterwijk E, Hulsbergen-Van de Kaa CA, Boerman OC, Oyen WJ, Langenhuijsen JF, Mulders PF. Tumor-targeted Dual-modality Imaging to Improve Intraoperative Visualization of Clear Cell Renal Cell Carcinoma: A First in Man Study. *Theranostics*. 10.7150/thno.23335 PMC5928878 29721070

[21] Sun M Shariat SF Trinh QD An evidence-based guide to the selection of sequential therapies in metastatic renal cell carcinoma. Ther Adv Urol 2013;5:121–8. 23554847 10.1177/1756287212466128 PMC3607488

[22] Guo J Jin J Oya M Safety of pazopanib and sunitinib in treatment-naive patients with metastatic renal cell carcinoma: Asian versus non-Asian subgroup analysis of the COMPARZ trial. J Hematol Oncol 2018;11:69. 29788981 10.1186/s13045-018-0617-1 PMC5964681

